# Detection of the neuropathogenic variant of equine herpesvirus 1 associated with abortions in mares in Poland

**DOI:** 10.1186/s12917-015-0416-7

**Published:** 2015-05-01

**Authors:** Karol Stasiak, Jerzy Rola, Gabor Ploszay, Wojciech Socha, Jan F Zmudzinski

**Affiliations:** Department of Virology, National Veterinary Research Institute, Al. Partyzantow 57, 24-100 Pulawy, Poland

**Keywords:** EHV-1, ORF30, Neuropathogenic genotype, Abortion

## Abstract

**Background:**

The incidence of reported cases of equine herpesvirus myeloencephalopathy (EHM) caused by infection with neuropathogenic strains of equine herpesvirus 1 (EHV-1) has markedly increased over the last decade in many Western countries. The purpose of this study was to estimate the prevalence of the neuropathogenic (*G2254*) and non-neuropathogenic (*A2254*) variants of EHV-1 among isolates associated with abortions in Polish stud farms.

**Results:**

The results of polymerase chain reaction-restriction fragment length polymorphism (PCR-RFLP) and sequencing were consistent, and showed that two out of 64 abortions (3.1%) were induced by the neuropathogenic genotype *G2254*. All remaining 18 EHV-1 positive abortion cases (28.1%) were caused by the non-neuropathogenic genotype *A2254*.

**Conclusions:**

Most of the abortions in mares in Poland from 1999 to 2012 were associated with non-neuropathogenic strains of EHV-1. However, the presented data indicate that the neuropathogenic genotype of the virus is also present in Polish stud farms. Such a presence suggests that the future emergence of EHM in Poland is probable.

## Background

Equine herpesvirus 1 (EHV-1) is a double-stranded DNA virus that occurs worldwide in all breeds of horses [1,2]. Infections caused by EHV-1 are important as clinical outbreaks of the disease still occur, despite preventive and control measures being taken [3,4]. Exposure to EHV-1 can cause upper respiratory tract infection in foals and young horses. In pregnant mares, the virus can be transferred across the uteroplacental barrier and infect the fetus, which can lead to late-gestation abortion. EHV-1 can also migrate with infected blood leukocytes to the central nervous system and replicate in endothelial cells of arterioles in the spinal cord and brain, causing vasculitis and thrombosis [5]; this syndrome is known as equine herpesvirus myeloencephalopathy (EHM). Previous research has shown that the neuropathogenicity of EHV-1 strains is strongly associated with a single point mutation in the open reading frame (ORF) 30 of the gene encoding viral DNA polymerase [6-8]. This mutation is a single nucleotide adenine to guanine substitution at nucleotide 2,254, corresponding to an asparagine to aspartic acid substitution. Additionally, two other studies revealed that another nucleotide substitution at nucleotide 2,258 of ORF30 could possibly be associated with neuropathogenicity [6,9].

Although the clinical form of EHM is less frequently observed than the other types of EHV-1 infection, it can cause serious economic losses in breeding horses and have a very negative impact on the functioning of veterinary hospitals, riding schools, and racetracks [4,3,10,11]. Moreover, recent data from the United States of America (US) showed that neuropathogenic strains of EHV-1 could become an important causative agent of abortions in mares even in the absence of actual clinical signs of EHM or respiratory disease [9].

In Poland, EHV-1 abortion outbreaks in mares have been reported several times since the early 1950s [12-15]. However, there are no data on the occurrence of neuropathogenic EHV-1 strains in Poland. The purpose of this study was to determine the prevalence of the neuropathogenic genotype of ORF30 among the strains of EHV-1 isolated from abortion cases in Poland.

## Methods

### Samples

We tested tissue samples (lung, liver, spleen, heart, kidney, and placenta, if delivered) from 64 aborted fetuses that were delivered to the Department of Virology of the National Veterinary Research Institute in Pulawy between 1999 and 2012. The whole fetuses or fetal organs came from horse studs located in different regions of Poland. None of the animals had been vaccinated against EHV-1, and none of the studs had a history of respiratory and neurological disease. Necropsy reports revealed that all 64 abortions occurred during the third trimester of pregnancy. A variety of macroscopic lesions were observed in most cases, including a large amount of pleural fluid, hepatic necrosis, and pulmonary oedema. No histological investigation was done. Organ samples from aborted fetuses were stored at −70°C until further processing.

Two grams of each tissue sample was used for preparation of 10% (w/v) suspension in Eagle’s Minimum Essential Medium (Sigma-Aldrich), supplemented with 1% antibiotic solution (Antibiotic Antimycotic Solution 100x, Sigma-Aldrich) using ULTRA-TURRAX® homogenizer. Tissue homogenates were centrifuged at 1,700 x g for 10 min, and then supernatants from the same fetus were pooled together and stored at −70°C until testing.

### DNA extraction

DNA was extracted from every pool of tissue supernatant using a phenol-chloroform-isoamyl alcohol mixture.

### PCR testing

The DNA obtained from tissue homogenates was tested for the presence of EHV-1 and EHV-4 using primers for glycoprotein B previously described by Kirisawa et al. (EHV1/4 Forward: 5′-CTT GTG AGA TCT AAC CGC AC-3′[1477-1496/1468-1487], EHV-1 Reverse: 5′-GCG TTA TAG CTA TCA CGT CC-3′[1936–1917], EHV-4 Reverse: 5′-CCT GCA TAA TGA CAG CAG TG-3′[2410–2391]) [16].

### Virus isolation

EHV-1 was isolated in 25-cm^2^ tissue culture flasks containing monolayers of RK13 cells. Flasks were inoculated and checked daily for appearance of cytopathic effect (CPE). CPE-positive flasks were frozen and stored at −70°C.

### Polymerase chain reaction-restriction fragment length polymorphism (PCR-RFLP)

PCR-RFLP neuropathogenic/non-neuropathogenic discrimination testing was performed on EHV-1-positive samples. PCR amplification of a 380-bp fragment of ORF30 was based on a modified protocol described by Allen [17]. A 25-μl reaction mix was prepared for PCR containing 0.05 U/μl AccuTaq LA DNA Polymerase, 200 μM of deoxynucleotide triphosphate mix, 1 × PCR buffer, 400 nM of the primer ORF30-Forward (5′-GTG GAC GGT ACC CCG GAC-3′[2005–2022]) and ORF30-Reverse (5′-GTG GGG ATT CGC GCC CTC ACC-3′[2384–2364]) and 2.5 μl DNA template, suspended in RNAse-DNAse-free water. The reaction was run in a Biometra Thermocycler (Biometra, Germany) under the following conditions: initial denaturation at 94°C for 3 min, followed by 35 cycles of denaturation at 94°C for 30 s, annealing at 60°C for 1 min, and elongation at 72°C for 30 s.

PCR products were digested with *Sal*I enzyme [recognition site: 5′…G↓TCGAC…3′] (EURx, Poland). Digestion was performed in a 50-μl reaction mixture containing 10 μl of PCR product, 5 μl 10x Buffer High, 0.5 μl 100x BSA (EURx), and 1 μl *Sal*I enzyme, suspended in nuclease free water. Digestion was run at 37°C for 2 h in a thermocycler. Products were visualized by electrophoresis on 1.5% agarose gel. DNA from two EHV-1 strains was used as a positive control: Ab4 (neuropathogenic strain) and V592 (non-neuropathogenic strain).

### Sequencing

All positive samples were confirmed by sequencing the 380-bp ORF30 fragment using the Sanger method at the Institute of Biochemistry and Biophysics Polish Academy of Science (Warsaw, Poland). Nucleotide sequences were assembled and aligned using Molecular Evolutionary Genetics Analysis (MEGA) version 5.0.5. The nucleotide sequences reported in this study were submitted to GenBank under the accession numbers KR080374-KR080393.

## Results

PCR analysis using Kirisawa’s PCR primers showed that 20 pooled samples were EHV-1 positive, but no samples were EHV-4 positive. Virus isolation was successful in all PCR positive samples, with a clearly visible CPE developing within 3–5 days after inoculation of cells (Table 1).

**Table 1:** **Polish ORF30 genotype of EHV-1 isolates by PCR-RFLP**

**Strain designation** ^**a**^	**Genotype at position 2254**	**Region** ^**b**^
PL/1999/I	A 2254	LU
PL/1999/II	A 2254	MA
PL/2001/I	A 2254	LU
PL/2002/I	A 2254	MA
PL/2003/I	A 2254	WP
PL/2004/I	A 2254	LU
PL/2004/II	A 2254	PM
PL/2005/I	A 2254	WM
PL/2006/I	A 2254	MP
PL/2006/II	A 2254	LU
PL/2007/I	A 2254	MA
PL/2008/I	A 2254	DS
PL/2009/I	A 2254	SL
PL/2009/II	G 2254	LU
PL/2010/I	A 2254	MA
PL/2010/II	G 2254	SL
PL/2010/III	A 2254	LU
PL/2011/I	A 2254	MP
PL/2012/I	A 2254	MA
PL/2012/II	A 2254	LU

^a^Based on the year of isolation.

^b^Voivodship SL-Silesian; MA-Masovian; WM-Warmian-Masurian.

LU-Lublin; MP-Lesser Poland; PM-Pomeranian; DS-Lower Silesian.

WP-Greater Poland. The regions are described in detail at:

http://en.wikipedia.org/wiki/Voivodeships_of_Poland.

Amplification using ORF30-specific PCR and further digestion of PCR products with *Sal*I enzyme showed that two of the 20 EHV-1 positive isolates were the neuropathogenic variant *G2254* (10% of EHV-1 positive isolates, and 3.1% of total abortion cases), whereas 18 were the non-neuropathogenic variant *A2254* (90% of EHV-1 positive isolates, and 28.1% of all abortion cases).

**Figure 1:**
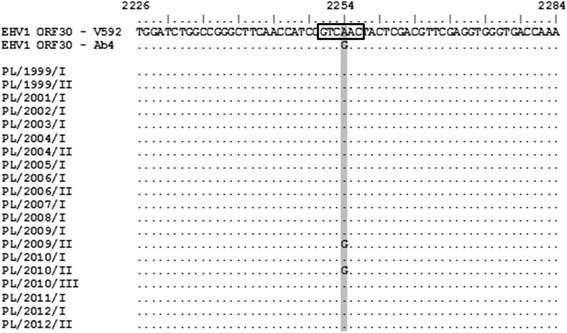
Sequence analysis of ORF30 of Polish EHV-1 isolates (PL/1999/I–PL/2012/II). Variable position 2,254 is shaded in the box marked restriction site *Sal*I enzyme.

Comparative nucleotide sequence analysis of the 380-bp fragment gene encoding the catalytic subunit (ORF30) of the viral DNA polymerase confirmed the presence of guanine at nucleotide position 2,254 in two isolates. The other EHV-1 isolates encoded adenine at the position 2,254 and were classified as non-neuropathogenic variants. No nucleotide substitution was found at position 2,258. The consensus alignment indicated that partial ORF30 sequences were identical to the sequences of appropriate reference strains of EHV-1: Ab4 (neuropathogenic) and V592 (non-neuropathogenic) (Figure 1).

## Discussion

There have been no previous reports of EHM outbreaks in Poland, and no potentially neuropathogenic variants of EHV-1 have previously been identified [18]. We have shown, for the first time, that the neuropathogenic genotype of EHV-1 circulates in the horse population in Poland. There was a clear predominance of the non-neuropathogenic (90% of EHV-1 positive cases) over the neuropathogenic EHV-1 genotype (10% of EHV-1 positive cases) as a causative agent of abortions in Polish stud farms. This proportion is similar to the results of recent studies in which abortion was associated with the neuropathogenic variant of EHV-1 in 0.9% of abortion cases in Japan [19], 7% in Argentina [20], 8.9% in Central Kentucky of the US [9], 10.6% in Germany [21], and 25.9% in France [22].

In contrast to a few abortion outbreaks in Argentina and Germany that were associated with neurological signs in mares, there were no clinical signs indicating EHM in any case of aborted fetuses tested in this study. These results are not unusual as previous studies have proved that although the presence of the neuropathogenic strain is a factor fostering an increase in EHM cases, it is not the only factor that determines the appearance of neurological disease in infected horses [23,24]. For example, a German study found that only two out of seven abortion cases caused by neuropathogenic EHV-1 strains were associated with EHM signs in pregnant mares [21]. Some studies have associated EHM with the presence of another substitution (adenine to cytosine) in ORF30 at the 2,258 position [9,21]; however, this was not detected in our study.

The EHV-1 isolates possessing a nucleotide substitution from A to G at the 2,254 position were detected in two distant provinces of Poland, hence it is unlikely that the abortions were caused by the same strain of the virus. As this study concentrated on abortion cases, only a fraction of the total EHV-1 infections in Poland were analyzed, whereas a previous study by Pronost et al. showed that the *G2254* genotype could also be associated with respiratory disease [22]. It is also possible that some of our cases were caused by mixed infection with two viral strains. Allen et al. described the occurrence of dual infections in the Thoroughbred broodmare population, with both neuropathogenic and non-neuropathogenic strains involved [25]. A similar situation took place in the case of horses infected after natural exposure at a racetrack in California [26]. Unfortunately, the diagnostic techniques used in our study were not able to detect simultaneous infection with both genotypes.

Finally, it cannot be excluded that EHM cases may have already appeared in Poland, but were either not reported or not identified properly by veterinarians. Even if this assumption is wrong, the fact that the *G2254* ORF30 variant of EHV-1 is present in the horse population means that the risk of EHM outbreaks in Poland should be taken into consideration.

## Conclusion

The presented data demonstrate that the neuropathogenic genotype of EHV-1 is present in Polish stud farms. Of the 20 EHV-1 abortion isolates, the vast majority belonged to the non-neuropathogenic marker *A2254* (18 out of 20 isolates, which was 90%), with only two out of the 20 isolates (10%) identified as the neuropathogenic genotype *G2254*. However, the presence of neuropathogenic EHV-1 strains in Polish studs suggests that the emergence of EHM cases in Poland is probable.

## Availability of supporting data

The data sets supporting the results of this article are included within the article.
